# Synergistic effects of grafting and companion cropping reshape the root endophytic microbiome network to regulate yield and fruit quality in continuously monocropped watermelon

**DOI:** 10.3389/fmicb.2026.1879144

**Published:** 2026-07-29

**Authors:** Liyun Kang, Xiaohui Li, Ningning Gao, Huiying Wang, Hailun Li, Jingjing Cheng, Yong Zhao, Gaozheng Chang, Ahmed Fathy Yousef, Fikru Tamiru Kenea, Weixing Zhao

**Affiliations:** 1 Horticultural Research Institute, Henan Academy of Agricultural Sciences, Zhengzhou, China; 2 Department of Horticulture, College of Agriculture, Al-Azhar University, Asyut, Egypt; 3 Department of Horticulture, College of Agriculture and Natural Resources, Dilla University, Dilla, Ethiopia

**Keywords:** *Citrullus lanatus*, companion cropping, continuous monocropping, co-occurrence networks, grafting, root endophytic microbiome, yield and fruit quality

## Abstract

**Introduction:**

Grafting and companion cropping alleviate continuous watermelon monocropping obstacles, yet their synergistic regulatory regulatory effects on root endophytic microbiota and fruit traits remain unclear.

**Methods:**

A 2 × 5 split-plot field trial was conducted in a 7-year continuous watermelon monocropping system, comprising grafted and non-grafted plants with five companion cropping treatments designated CK, T1, T2, T3, and T4. High-throughput sequencing, PCoA, PERMANOVA, RDA, microbial co-occurrence network analysis and yield-quality assays were applied for systematic characterization.

**Results:**

Grafting significantly increased total bacterial OTUs, all bacterial richness and diversity indices, fungal OTU numbers and fungal richness, yet reduced fungal community diversity. PERMANOVA verified that grafting, companion cropping and their interaction significantly reshaped bacterial and fungal community structures (*p* < 0.001). Companion cropping reduced unique microbial OTUs and remodeled community composition and network topology. Under grafting conditions, companion cropping constructed tighter and more complex microbial networks and alleviated interspecies competition. LEfSe analysis demonstrated that grafting induced phylum-level differentiation of bacterial and fungal signature taxa, whereas companion cropping primarily modulated low-abundance and rare microbial assemblages. Grafting elevated yield titratable acidity and vitamin C while decreasing soluble sugar and soluble protein. Companion cropping raised soluble protein and vitamin C in grafted watermelon and improved yield, soluble sugar, soluble protein and vitamin C in non-grafted watermelon (*p* < 0.05). All companion treatments significantly reduced titratable acidity (p < 0.05). RDA clearly separated grafted and non-grafted microbiota along the first axis and revealed directional correlations between dominant phyla and fruit traits, which were confirmed by Pearson correlation analysis.

**Discussion:**

Combined grafting and companion cropping optimize watermelon yield and nutritional quality by restructuring the root endophytic microbial communities, offering a feasible cultivation strategy for sustainable continuous watermelon production.

## 1 Introduction

Watermelon (*Citrullus lanatus*, Cucurbitaceae) is an economically important crop worldwide, with China as the largest producer and consumer. In 2024, China’s watermelon cultivation area reached 1.452 million hectares and produced 63.51 million tons, accounting for 49.12 and 60.94% of the global totals, respectively (FAOSTAT, http://faostat.fao.org). To meet market demand, watermelon production has increasingly shifted toward large-scale, continuous monocropping systems (Gu et al., 2022; Tong et al., 2025).

However, long-term monocropping disrupts soil microbial balance, enriching soil-borne pathogens while depleting beneficial microbes, a shift closely linked to yield decline and quality deterioration (Ghani et al., 2022; He et al., 2025; Liu P. F. et al., 2025; Guo et al., 2025). Root-associated microbiomes, particularly endophytes, are critical for plant growth and stress resistance, and thus play a key role in alleviating monocropping-induced soil degradation (Marie et al., 2025; Cheng et al., 2024).

Unlike rhizosphere and bulk soil microbes susceptible to environmental fluctuation, root endophytes reside inside root tissues and form intimate, stable associations with host plants (Wilson et al., 1995; Edwards et al., 2015). They are recruited from the rhizosphere and shaped by host genotype and microbial interactions. Functionally, root endophytes enhance plant growth by improving nitrogen fixation capacity (Cooney et al., 2025) and facilitating the uptake of essential nutrients, including nitrogen, phosphorus, and potassium (Papri et al., 2024; Kannaiah et al., 2023). In addition, they contribute to stress adaptation by modulating host metabolic pathways (Zhou et al., 2025) and promoting the production of bioactive secondary metabolites (Etalo et al., 2018), thereby enhancing plant resilience under adverse environmental conditions (Santamaria et al., 2017). Despite their functional importance, most monocropping studies have focused on rhizosphere or bulk soil microbiomes, leaving root endophytic communities largely underexplored.

Grafting and companion cropping are two effective strategies to mitigate monocropping constraints (Goldschmidt et al., 2014; Mehdi et al., 2025). Grafting enhances stress tolerance via scion–rootstock interactions (Qin et al., 2024; Tomo et al., 2025; Noémi et al., 2024). Companion cropping improves the soil microenvironment, nutrient use efficiency and microbial diversity (Xu et al., 2015; Haile et al., 2023). While each practice individually shapes soil microbiomes, how their interactive effects specifically target root endophytic communities, rather than bulk soil or rhizosphere microbes, and whether these shifts affect yield and quality remain unclear.

We propose two testable hypotheses: (1) grafting and companion cropping synergistically alter the diversity and composition of watermelon root endophytic bacterial and fungal communities; and (2) these reshaped endophytic communities enhance nutrient-related microbial functions, thereby improving watermelon yield and fruit quality under long-term monocropping. Therefore, this study provides a theoretical framework for understanding how grafting–companion cropping systems reshape root-associated microecological processes. It also offers practical insights for the development of sustainable management strategies and microbial-based technologies aimed at alleviating continuous watermelon monocropping constraints.

## 2 Materials and methods

### 2.1 Test materials

The small-fruited watermelon cultivar ‘SWT No.2’ was provided by the Institute of Horticulture, Henan Academy of Agricultural Sciences. The chive (*Allium schoenoprasum*) and onion (*Allium cepa*) cultivars used were local varieties purchased from the Chenzhai Flower Market in Zhengzhou City.

### 2.2 Experimental site and experimental design

The experiment was conducted at the Modern Agricultural Science and Technology Experiment and Demonstration Base of Henan Academy of Agricultural Sciences (35°45′N, 113°37′E). The site is located in a warm temperate continental monsoon climate zone, with an average annual temperature of approximately 14 °C. The soil is classified as fluvo-aquic soil.

The experimental field covered an area of 150 m^2^ and was arranged in a split-plot design. Grafting status served as the main-plot factor, including grafted watermelon (G, using zucchini *Cucurbita pepo* L. as rootstock) and non-grafted watermelon (NG). The field had been continuously cultivated with watermelon since 2018, resulting in a 7-year continuous monocropping history by 2024.

From March to June 2025, five treatments were established within both the grafted (G) and non-grafted (NG) zones, including the control watermelon monocropping (CK), watermelon companion cropped with chive on one side (T1), chive on both sides (T2), onion on one side (T3) and onion on both sides (T4). Each treatment was arranged in a randomized block design with three replications. Field management practices, such as irrigation and weeding, were kept consistent throughout the watermelon growth period. Watermelon seeds were sown in seedling trays and transplanted at the three-leaf stage with a row spacing of 110 cm and a plant spacing of 35 cm. One week after watermelon transplantation, chive or onion bulbs were planted at a distance of 5–8 cm from each watermelon seedling, with a planting spacing of 12 cm for both chive and onion.

### 2.3 Sample collection and measurements

#### 2.3.1 Root sample collection and processing

During the fruit swelling stage of watermelon, three plants with uniform growth were selected from each plot, with a total of 90 plants. The soil within an approximate 25 cm radius around each plant was carefully loosened with a sterilized iron shovel, and the entire plant was then gently uprooted by hand from the base of the stem. Loosely adhering soil was removed by gently shaking, and the root samples were immediately transported to the laboratory in an ice box. For the endophytic microbial analysis, root surface sterilization was performed as described by Ren et al. (2019) with minor modifications. Root samples were placed in 50 mL centrifuge tubes, immersed in sterile water, and then shaken at 180 rpm for 30 min to remove loosely attached soil particles. Afterwards, the roots were sequentially immersed in 75% ethanol for 30 s, 2.5% sodium hypochlorite (containing 0.1% Tween 80) for 5 min, and 75% ethanol for 30 s. Finally, the roots were rinsed five times with sterile distilled water to remove residual sterilizing agents. To confirm the efficacy of the surface sterilization, 100 μL aliquots from the final rinse water were plated on R2A and potato dextrose agar (PDA) plates and incubated at 28 °C for 7 days; no colony growth was observed for any sample. The processed root samples were stored at −80 °C for the analysis of their endophytic microbial community structures.

#### 2.3.2 Yield and fruit quality determinations

At fruit maturity, 10 representative plants were selected from each plot to determine the fruit weight per plant, and the yield was calculated based on the plot area (300 plants in total). Three fruits were randomly selected from each plot for quality index determination. Soluble sugar content was measured using the anthrone colorimetric method; titratable acidity was measured using the NaOH titration method; soluble protein content was assayed using the Coomassie Brilliant Blue G-250 staining method; and vitamin C content was quantified using the 2,6-dichloroindophenol titration method (Li, 2000).

### 2.4 Total DNA extraction, PCR amplification, and sequencing of root samples

Total DNA extraction, PCR amplification, and sequencing of root samples were conducted by Beijing Biomarker Technologies Corporation. Genomic DNA was extracted using a Genomic DNA Extraction Kit (Tiangen Biotech, Beijing, China), following the manufacturer’s instructions, with all centrifugation steps performed at 12,000 rpm (~13,400 × g). DNA concentration and purity were determined using a microplate reader (Synergy HTX, BioTek Instruments, USA). PCR amplification was performed on a gradient thermal cycler (Veriti 96-Well Thermal Cycler, Applied Biosystems, USA). The V3 + V4 hypervariable region of the bacterial 16S rRNA gene was amplified by primers 335F (5’-CADACTCCTACGGGAGGC-3′) and 769R (5’-ATCCTGTTTGTMMTCCCCVCRC-3′). The fungal ITS1 region was amplified using primer pair ITS1F (5’-CTTGGTCATTTAGAGGAAGTAA-3′) and ITS2 (5’-GCTGCGTTCTCTCATCGATGC-3′). All purified PCR amplicons were subjected to high-throughput sequenceing on the Illumina NovaSeq 6000 platform with PE150 read length. The PCR products were verified by 1.8% agarose gel electrophoresis to detect their integrity. PCR products with good integrity were used for library construction with the KOD FX Neo Library Construction Kit (TOYOBO, Japan). Sequencing was performed on the Illumina NovaSeq 6000 platform (Illumina, USA).

Raw sequencing data were demultiplexed and quality-filtered using QIIME2 (version 2023.2; Bolyen et al., 2019) with the following criteria: reads with an average quality score < Q20 over a 5-bp sliding window were truncated, and reads shorter than 150 bp after trimming were discarded. Chimeric sequences were detected and removed using the UCHIME algorithm (reference-based mode against the Silva 138.1 database for bacteria and the UNITE 9.0 database for fungi). Clean reads were clustered into operational taxonomic units (OTUs) at a 97% sequence similarity threshold using UPARSE (version 11.0.667). The most abundant sequence within each OTU was selected as the representative sequence for downstream analyses.

The raw sequencing data have been deposited in the NCBI SRA under BioProject accession number PRJNA1462526. The data are currently private and will be released upon publication. Access can be requested via NCBI.

Taxonomic annotation of bacterial OTUs was performed against the Silva database (Release 138.1) using the RDP classifier with a confidence threshold of 0.8. Fungal OTUs were annotated against the UNITE database (version 9.0, released February 2023) using BLAST with an E-value cutoff of < 1 × 10^−5^. Kingdom-only OTUs without phylum assignments were discarded to remove sequencing false positives. QC-passed OTUs with clear kingdom matches but no known phyla, due to incomplete database coverage, were kept as unclassified_Bacteria or unclassified_Fungi. The OTU abundance matrix obtained through the above-described bioinformatics quality control procedures was directly used for diversity analyses (including *α*- and *β*-diversity). ACE and Chao1 indices estimate microbial speches richness for both bacteria and fungi, whereas the Shannon and Simpson indices quantify overall diversity by jointly accounting for both richness and evenness. Notably, the Shannon index is more sensitive to low-abundance taxa, whereas the Simpson index places greater weight on dominant, high-abundance taxa.

### 2.5 Statistical analyses

Microbial diversity analyses were conducted using the BMKCloud platform (^1^), including the rarefaction curve generation, Venn diagram construction, *α*-diversity index calculation, principal coordinate analysis (PCoA), redundancy analysis (RDA) and visualization of root endophytic microbial taxonomic composition at the phylum and genus levels. To formally assess the enrichment or depletion of taxonomic groups across treatments, we further conducted LEfSe analysis (Segata et al., 2011) (LDA scores > 3.5, *p* < 0.05). Bar charts illustrating watermelon yield and fruit quality parameters were generated using the MetWare Cloud Online Analysis System.^2^

Split-plot ANOVA was performed using the Data Processing System (DPS; Tang et al., 2013) with grafting (G/NG) as the main-plot factor and companion cropping (CK, T1–T4) as the subplot factor. This model was used to test the main effects of grafting and companion cropping, as well as their interaction (G × C), on root endophytic α-diversity indices, watermelon yield, and fruit quality traits. Duncan’s multiple range test was used for post-hoc comparisons, and differences with *p* < 0.05 were considered statistically significant. When a significant interaction was detected, simple effects analyses were conducted to compare companion cropping treatments separately within each grafting condition. For root endophytic community structure (β-diversity), Bray-Curtis distance matrices were constructed on the BMKCloud platform. Community differences were tested by PERMANOVA (adonis2, 9,999 permutations) on the corresponding distance matrices (Oksanen et al., 2026), evaluating grafting, companion cropping, and their interaction (*R^2^* and *p* reported). Redundancy analysis (RDA) was performed with permutation tests (9,999 permutations) used to evaluate the significance of the RDA axes. Pearson’s correlation coefficients were used to examine the relationships between yield and fruit quality traits and root endophytic microbial communities at both phylum and genus levels, with results visualized as heatmaps.

Prior to co-occurrence network construction, this matrix was further filtered to exclude OTUs with relative abundance < 0.01% or those detected in fewer than 3 samples (prevalence < 33.3%), thereby reducing spurious correlations that might be caused by rare OTUs. Each network was constructed using the Sparse Correlations for Compositional data (SparCC) algorithm (Friedman and Alm, 2012) based on the 9 biological replicates in each treatment. As noted in methodological studies, networks inferred from only 9 samples per treatment have limited reliability, and therefore all network results should be interpreted as exploratory (Kajihara and Hynson, 2024; Kurtz et al., 2015). Co-occurrence network analysis was performed based on Spearman’s rank correlation coefficients, calculated via SparCC to account for the compositional nature of sequencing data. Only robust correlations with |*r*| > 0.8 and *p* < 0.05, adjusted using the Benjamini–Hochberg false discovery rate (Caldas de Castro et al., 2006) were retained. Networks were constructed and visualized using Gephi (version 0.10.1) (Bastian et al., 2009), where node colors represent taxonomic groups at the phylum level and node sizes correspond to relative abundance. Following standard microbial network comparison procedures, we calculated network topological properties, including network density, average clustering coefficient, average path length, and modularity, to compare treatment effects (Liu et al., 2023).

All figures were further refined using Adobe Illustrator 2024 (Adobe Inc., San Jose, CA, USA) for layout optimization, color standardization, and graphical consistency.

## 3 Results

### 3.1 Endophytic bacterial diversity in watermelon roots

#### 3.1.1 Bacterial OTU analysis

**Figure 1:**
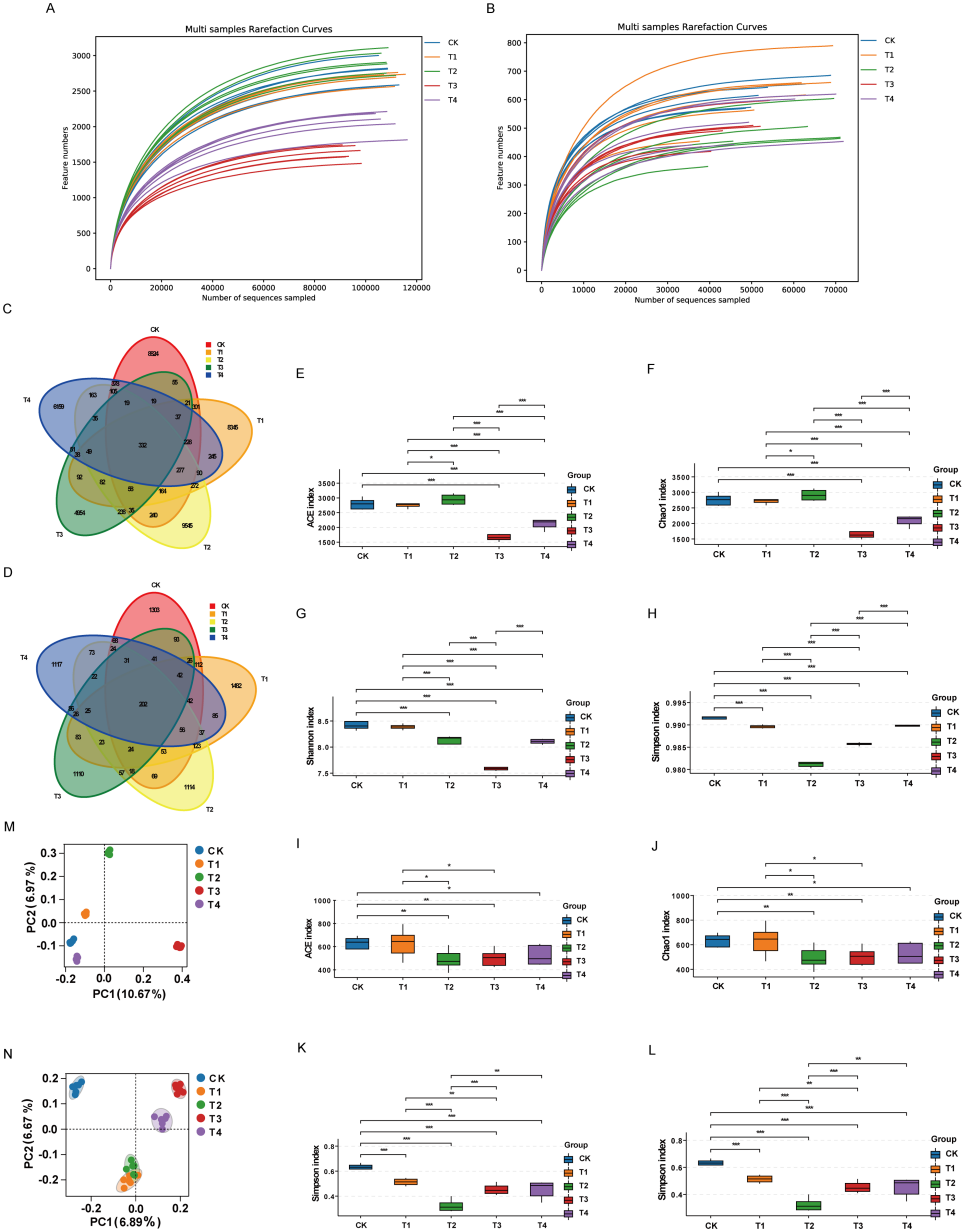
Analysis of bacterial communities in grafted and non-grafted watermelon roots. **(A)** Rarefaction curve of bacterial operational taxonomic units (OTUs) in grafted watermelon. **(B)** Rarefaction curve in non-grafted watermelon. **(C)** Venn diagram of shared OTUs in grafted group. **(D)** Venn diagram in non-grafted group. **(E–H)** Diversity indices (ACE, Chao1, Shannon, Simpson) in grafted group. **(I–L)** Diversity indices in non-grafted group. **(M)** Principal coordinate analysis (PCoA) of grafted group. **(N)** PCoA of non-grafted group. *, **, and *** denote significant differences at the *p* < 0.05, *p* < 0.01, and *p* < 0.001 levels, respectively.

High-throughput sequencing identified 41,451 bacterial OTUs in grafted watermelon roots and 11,093 OTUs in non-grafted roots (Figure 1). Rarefaction curves approached asymptotes for both treatments (Figures 1A,B), confirming sufficient sequencing depth to characterize core endophytic bacterial community structures.

Venn diagram analysis revealed the distribution of shared and unique OTUs among treatments (Figures 1C,D). Under grafted conditions, only 332 OTUs were shared among all five treatments (Figure 1C), with companion cropping treatments (except T2) exhibiting fewer unique OTUs than the control (CK). Similarly, under non-grafted conditions, only 202 OTUs were common to all treatments, and companion cropping treatments (except T1) showed reduced unique OTU numbers relative to CK (Figure 1D).

#### 3.1.2 Bacterial community richness and diversity

Sequencing coverage exceeded 0.99 across all treatments, confirming the adequacy and representativeness of the *α*-diversity estimates. Split-plot ANOVA revealed significant main effects of grafting, companion cropping and their interactions on all four α-diversity indices (Table 1, *p* < 0.05). Given the significant interaction, simple effects of companion cropping were analyzed separately within each grafting status.

**Table 1:** ANOVA results for *α*-diversity metrics of root endophytic microbial communities

Microbial community	Source of variation	*F* value			
		ACE	Shannon	Simpson	Chao1
Bacterial	Grafting (G)	4823.492**	11205.962**	4657.728**	4656.474**
	Companion cropping (C)	89.546**	73.625**	48.869**	88.312**
	G × C	70.963**	35.689**	42.763**	69.723**
Fungal	Grafting (G)	874.766**	11.33**	155.923**	847.442**
	Companion cropping (C)	17.975**	10.337**	11.294**	17.730**
	G × C	3.341*	4.187**	12.286**	3.290*

G: grafting treatment; C: companion cropping treatment; G × C: interaction between grafting and companion cropping. *P < 0.05; **p < 0.01.

Grafting significantly increased all four α-diversity indices (ACE, Chao1, Shannon and Simpson) compared to non-grafted watermelon roots (Figures 1E–L; *p* < 0.05), demonstrating that grafting markedly enhanced both richness and diversity of endophytic bacterial communities. Companion cropping, conversely, reduced all four α-diversity indices relative to CK regardless of grafting status. Specifically, richness indices (ACE and Chao1) were lowest in T3, whereas diversity indices (Shannon and Simpson) were minimized in both T2 and T3 (Figures 1E–L; *p* < 0.05). Notably, grafting amplified the suppressive effects of companion cropping, as evidenced by more pronounced reductions in Shannon and Simpson indices under T2 and T3 in grafted versus non-grafted roots.

Principal coordinates analysis (PCoA) at the OTU level revealed distinct clustering patterns among treatments under both grafting conditions (Figures 1M,N). The first two principal coordinates explained 10.67 and 6.97% of total variation under grafted conditions and 6.89 and 6.67% under non-grafted conditions. PERMANOVA further confirmed that grafting, companion cropping, and their interaction all significantly shaped root endophytic bacterial community structure (*p* < 0.001, Table 2).

**Table 2:** PERMANOVA results based on Bray-Curtis distance for root endophytic microbial community composition

Microbial community	Source of variation	df	SS	MS	F value	R^2^	*p* value
Bacterial	Grafting (G)	1	8.8909	8.8909	1222.95	0.7770	0.001***
	Companion cropping (C)	4	1.0771	0.2693	37.04	0.0941	0.001***
	G × C	4	1.1105	0.2776	38.19	0.0971	0.001***
	Residuals	50	0.3635	0.0073	0.0318		
Fungal	Grafting (G)	1	6.1392	6.1392	537.86	0.7056	0.001***
	Companion cropping (C)	4	0.9992	0.2498	21.89	0.1148	0.001***
	G × C	4	0.9917	0.2479	21.72	0.1140	0.001***
	Residuals	50	0.5707	0.0114	0.0656		

G: grafting treatment; C: companion cropping treatment; G × C: interaction between grafting and companion cropping. df, degrees of: freedom; SS: sum of squares; MS: mean square; R^2^: proportion of explained community variation; P value: significance assessed via 9,999 permutations. ***p < 0.001.

#### 3.1.3 Bacterial community composition

**Figure 2:**
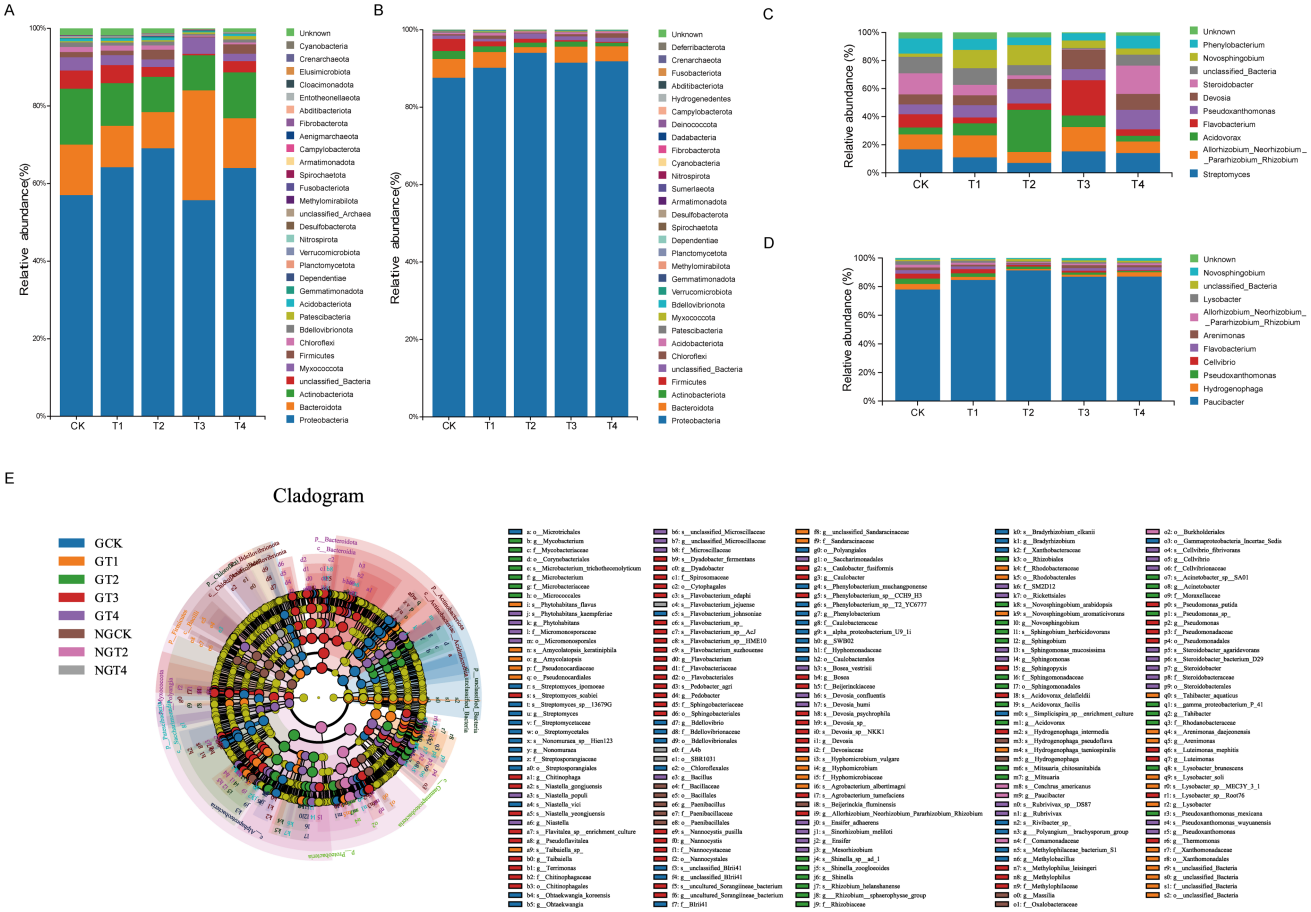
Composition of bacterial communities in watermelon roots under different grafting and companion cropping treatments. **(A)** Phylum-level bacterial community composition in grafted watermelon. **(B)** Phylum-level bacterial community composition in non-grafted watermelon. **(C)** Genus-level bacterial community composition in grafted watermelon. **(D)** Genus-level bacterial community composition in non-grafted watermelon. **(E)** Phylum-level bacterial biomarkers identified by LEfSe (LDA > 3.5). G: grafted; NG: non-grafted. CK: monocropping control; T1–T4: companion cropping treatments.

To investigate how significant grafting × companion cropping interactions affect specific taxonomic groups, the relative abundances of dominant bacterial phyla and genera are described below (Figures 2A–D). To formally assess the enrichment or depletion of taxonomic groups across treatments, we further conducted LEfSe analysis (LDA scores > 3.5, *p* < 0.05). The cladogram in Figure 2E visually illustrates the phylogenetic distribution of bacterial taxa that showed significant differentiation across the 10 treatment groups. At this screening threshold, each treatment group possessed its own distinctive signature taxa.

Grafted and non-grafted systems exhibited distinct bacterial community differentiation. In grafted systems, all signature taxa were associated exclusively with core bacterial phyla. CK was characterized by Bacteroidota. T1 was characterized by Firmicutes. T2 was characterized by Chloroflexi. T3 was characterized by Proteobacteria. T4 was characterized by Actinobacteriota. In non-grafted systems, while the same phyla were present, their treatment associations differed. CK was dominated by Proteobacteria. T2 was characterized by Bacteroidota. T4 was characterized by Actinobacteriota. Notably neither T1 nor T3 yielded phylum-level signature taxa. However, numerous distinct differentially abundant clades were detected at the genus and species levels, indicating that these two treatments primarily regulate the root bacterial community by modulating low-abundance and rare bacterial assemblages.

At the phylum level, grafting expanded phylum-level diversity, with 32 bacterial phyla detected in grafted roots versus 13 in non-grafted roots (Figures 2A,B). Four phyla were dominant (relative abundance > 1%) in both systems, namely *Proteobacteria*, *Bacteroidota*, *Actinobacteriota*, and *Firmicutes*. The corresponding values were 62.01, 14.84, 11.07, and 1.53% in grafted roots versus 91.03, 3.67, 1.39, and 1.20% in non-grafted roots, respectively. Under grafted conditions, companion cropping induced treatment-specific shifts. T1, T2, and T4 increased *Proteobacteria* while decreasing *Bacteroidota* and *Myxococcota.* Conversely, T3 enriched both *Bacteroidota* and *Myxococcota*. All companion treatments (except T1) reduced unclassified_Bacteria, and all reduced *Actinobacteriota* relative to CK (Figure 2A). Under non-grafted conditions, companion cropping consistently decreased *Bacteroidota*, *Actinobacteriota*, and *Firmicutes* while increasing *Proteobacteria* compared to CK (Figure 2B).

At the genus level, taxonomic analysis revealed distinct dominant endophytic bacterial genera in grafted versus non-grafted watermelon roots. In grafted roots, the top 10 genera excluding “Others” and unknown were *Streptomyces*, *Allorhizobium–Neorhizobium–Pararhizobium–Rhizobium*, *Acidovorax*, *Flavobacterium*, *Pseudoxanthomonas*, *Devosia*, *Steroidobacter*, unclassified_Bacteria, *Novosphingobium*, and *Phenylobacterium* (Figure 2C). In non-grafted roots, *Paucibacter*, *Hydrogenophaga*, *Pseudoxanthomonas*, *Cellvibrio*, *Flavobacterium*, *Arenimonas*, *Allorhizobium–Neorhizobium–Pararhizobium–Rhizobium*, *Lysobacter*, unclassified_Bacteria, and *Novosphingobium* dominated (Figure 2D). Five genera were shared by both grafting systems, namely *Allorhizobium–Neorhizobium–Pararhizobium–Rhizobium*, *Flavobacterium*, *Pseudoxanthomonas*, unclassified_Bacteria, and *Novosphingobium*. Their relative abundances were 4.55, 3.64, 3.60, 3.03 and 2.95% in grafted roots, respectively, compared with 1.72, 1.45, 1.05, 0.99 and 0.99% in non-grafted roots. Under grafted conditions, companion cropping increased *Streptomyces*, *Pseudoxanthomonas* and *Novosphingobium* while decreasing unclassified_Bacteria and *Phenylobacterium*. Most treatments (except T3) reduced *Flavobacterium*, and most (except T4) increased *Acidovorax* (Figure 2C). Under non-grafted conditions, companion cropping elevated *Paucibacter* but suppressed *Hydrogenophaga*, *Pseudoxanthomonas*, *Cellvibrio*, *Flavobacterium*, *Allorhizobium*–*Neorhizobium*–*Pararhizobium*–*Rhizobium*, and *Lysobacter* relative to CK (Figure 2D). Most treatments (except T1) increased unclassified_Bacteria.

### 3.2 Endophytic fungal community diversity in watermelon roots

#### 3.2.1 Fungal OTU analysis

**Figure 3:**
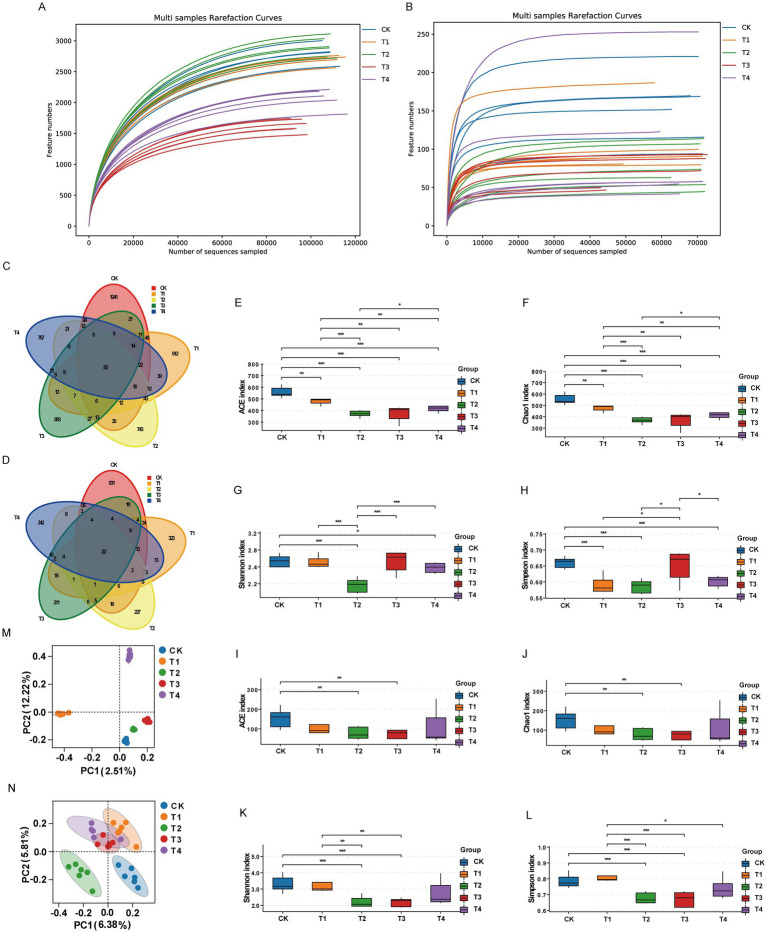
Analysis of fungal communities in grafted and non-grafted watermelon roots. **(A)** Rarefaction curve of fungal OTUs in grafted watermelon. **(B)** Rarefaction curve in non-grafted watermelon. **(C)** Venn diagram of shared OTUs in grafted group. **(D)** Venn diagram in non-grafted group. **(E – H)** Diversity indices (ACE, Chao1, Shannon, Simpson) in grafted group. **(I–L)** Diversity indices in non-grafted group. (M) Principal coordinate analysis (PCoA) of grafted group. **(N)** PCoA of non-grafted group. *, **, and *** denote significant differences at the *p* < 0.05, *p* < 0.01, and *p* < 0.001 levels, respectively.

High-throughput sequencing identified 5,068 fungal OTUs in grafted roots and 1,882 OTUs in non-grafted roots (Figure 3). Rarefaction curves approached saturation for both treatments (Figures 3A,B), confirming adequate sequencing depth.

Venn diagram analysis showed that companion cropping significantly reduced unique fungal OTUs relative to CK regardless of grafting status (Figures 3C,D). Only 82 OTUs were shared among the five grafted treatments, and only 22 OTUs were shared among non-grafted treatments.

#### 3.2.2 Fungal community richness and diversity

Sequencing coverage exceeded 0.99 across all treatments, confirming the adequacy and representativeness of the *α*-diversity estimates. Split-plot ANOVA revealed significant main effects of grafting, companion cropping, and their interactions on all four α-diversity indices (Table 1, *p* < 0.05). Given the significant interaction, simple effects of companion cropping were analyzed separately within each grafting status.

Grafting significantly increased fungal community richness (ACE and Chao1 indices; Figures 3E,F,I,J; *p <* 0.05) but decreased diversity (Shannon and Simpson indices; Figures 3G,H,K,L; *p <* 0.05) compared to non-grafted roots.

Companion cropping significantly reduced ACE and Chao1 indices under both grafting conditions, with T2 and T3 showing the lowest values (Figures 3E,F,I,J; *p <* 0.05). Effects on Shannon and Simpson indices were grafting-dependent: under grafted conditions, T1, T2, and T4 significantly reduced Simpson indices, and T2 and T4 reduced Shannon indices, with T2 showing the strongest inhibition (Figures 3G,H). Under non-grafted conditions, T2 and T3 significantly decreased both indices (Figures 3K,L).

PCoA revealed distinct fungal community clustering among treatments (Figures 3M,N). The first two principal coordinates explained 12.22 and 2.51% of variation under grafted conditions and 6.38 and 5.81% under non-grafted conditions. PERMANOVA further confirmed that grafting, companion cropping, and their interaction all significantly shaped root endophytic fungal community structure (*p* < 0.001, Table 2).

#### 3.2.3 Fungal community composition

**Figure 4:**
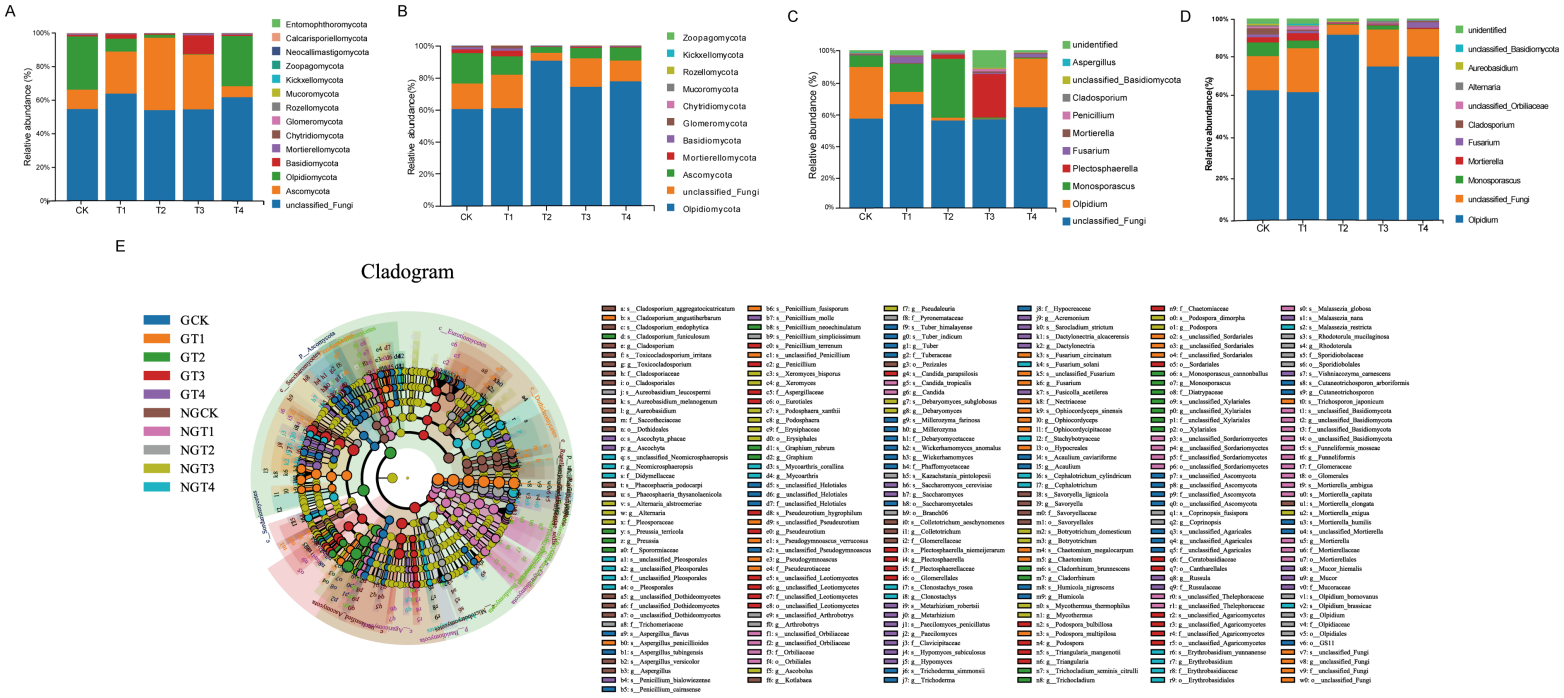
Composition of fungal communities in watermelon roots at the phylum and genus levels. **(A)** Phylum-level fungal community composition in grafted watermelon. **(B)** Phylum-level fungal community composition in non-grafted watermelon. **(C)** Genus-level fungal community composition in grafted watermelon. **(D)** Genus-level fungal community composition in non-grafted watermelon. **(E)** Phylum-level fungal biomarkers identified by LEfSe (LDA > 3.5). G: grafted; NG: non-grafted. CK: monocropping control; T1–T4: companion cropping treatments.

To examine how the significant grafting × companion cropping interaction shaped specific taxa, the relative abundances of dominant fungal phyla and genera are described below (Figures 4A–D). To formally assess taxon enrichment or depletion among treatments, we further performed LEfSe analysis (LDA score > 3.5, *p* < 0.05). The resulting cladogram in Figure 4E visualizes the phylogenetic distribution of significantly differentiated fungal taxa across the 10 treatment groups. Under this screening threshold, each treatment group was characterized by its own set of signature taxa.

Grafting and non-grafting systems exhibited pronounced fungal community differentiation. In the grafting system, all signature taxa were exclusively affiliated with *Ascomycota*. Under grafted conditions, CK was enriched in *Cladosporium*. T1 was enriched in *Penicillium*. T2 was enriched in *Plectosphaerella*. T3 was enriched in *Hypomyces*. T4 was enriched in *Monosporascus cannonballus*. By contrast, the non-grafting system was characterized primarily by *Olpidiomycota* and *Mortierellomycota* as the core discriminative phyla. Under non-grafted conditions, CK was dominated by indigenous *Olpidiomycota* taxa. T1 featured *Mortierella* as the signature genus. T2 was enriched in *Olpidium*. T4 was enriched in *Podospora*. Notably, T3 under non-grafted conditions yielded no phylum-level signature taxon. However, numerous distinct differentially abundant clades were detected at the genus and species levels, indicating that this treatment primarily modulates the root fungal community by reshaping low-abundance and rare fungal assemblages.

At the phylum level, four dominant fungal phyla (relative abundance > 1%) were identified in both grafting systems, with three shared, namely Olpidiomycota, unclassified_Fungi and Ascomycota (Figures 4A,B). Their relative abundances differed substantially. The corresponding values were 14.26, 57.86, and 23.80% in grafted roots versus 72.77, 14.51 and 9.74% in non-grafted roots, respectively. Under grafted conditions, T1, T2 and T3 increased *Ascomycota* while decreasing *Olpidiomycota* relative to CK (Figure 4A). Both T1 and T3 enriched *Basidiomycota*, while unclassified_Fungi remained unchanged across treatments. Under non-grafted conditions, companion cropping consistently reduced *Ascomycota* compared to CK (Figure 4B). T2, T3, and T4 increased *Olpidiomycota* but decreased *Mortierellomycota*, whereas T1 enriched *Mortierellomycota*. T1 and T3 increased unclassified_Fungi, while T2 decreased it.

At the genus level, five dominant fungal genera (relative abundance > 1%, excluding “Others”) were identified, with four shared between grafting systems, namely unclassified_Fungi, *Olpidium*, *Monosporascus* and *Fusarium* (Figures 4C,D). Their relative abundances were 57.86, 14.26, 12.51 and 1.43% in grafted roots, versus 14.51, 72.77, 2.46 and 1.12% in non-grafted roots, respectively. Under grafted conditions, T1, T2 and T3 reduced the pathogenic genus *Olpidium* (Figure 4C). *Monosporascus* was elevated by T1 and T2 but suppressed by T3 and T4. Unclassified_Fungi showed limited variation across treatments (Figure 4C). Under non-grafted conditions, companion cropping (except T1) increased *Olpidium* while all treatments reduced *Monosporascus* (Figure 4D). *Mortierella* decreased under T2–T4 but increased under T1. For Fusarium specifically, *Fusarium* was reduced by T1–T3 but elevated by T4. Unclassified_Fungi increased under T1 and T3 but decreased under T2 and T4.

### 3.3 Co-occurrence network analysis of root Endophytic microbial communities

**Figure 5:**
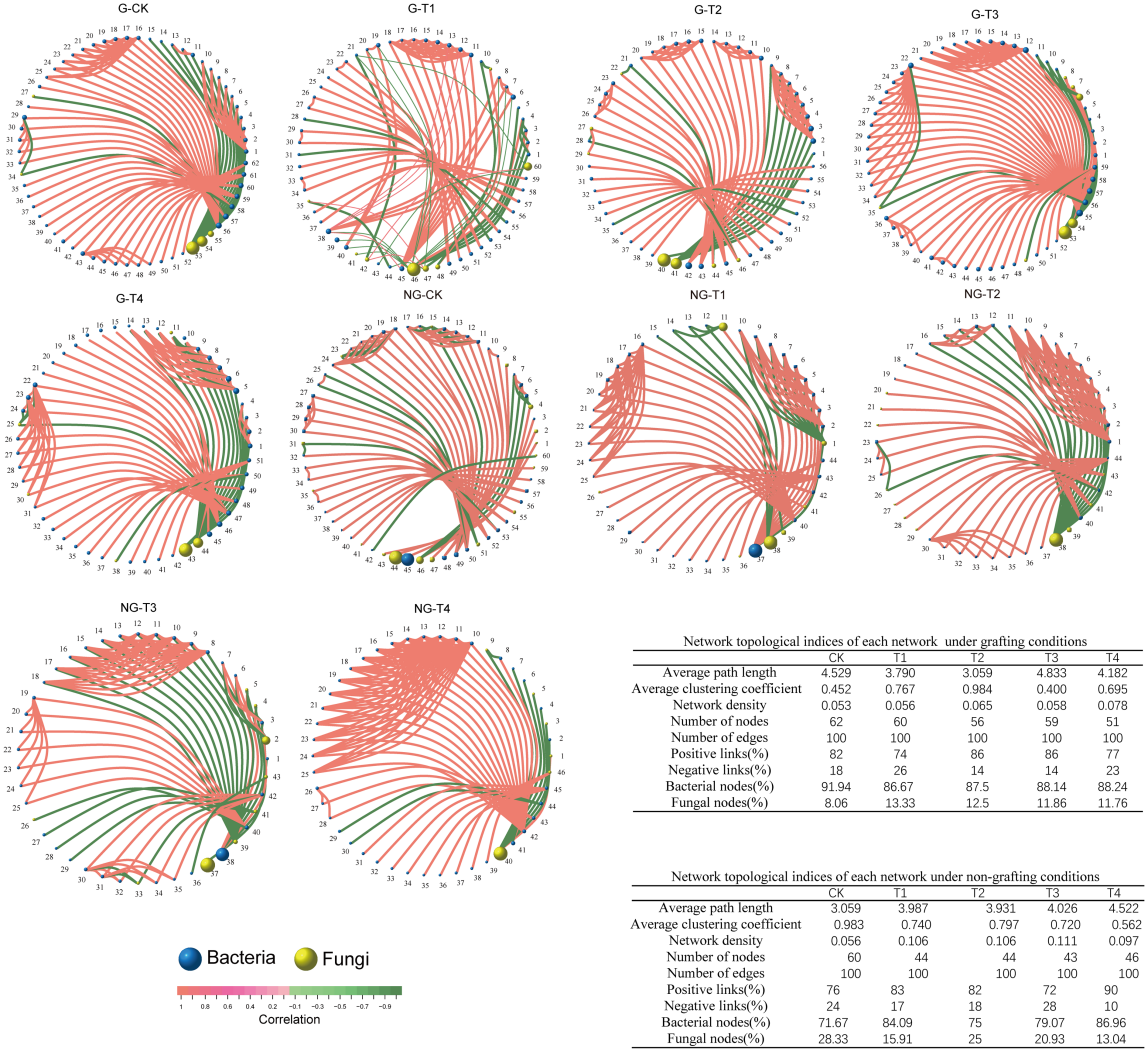
Co-occurrence network interactions between root endophytic bacteria and fungi in grafted (G) and non-grafted (NG) watermelon under different companion cropping treatments. The table shows the topological indices of each co-occurrence network.

Grafting status exerted a pronounced modulatory effect on the restructuring of endophytic microbial co-occurrence networks in watermelon roots in response to companion cropping. This included changes in their topological properties and interspecific interaction patterns. As shown in Figure 5, companion cropping markedly restructured these network properties under both grafted and non-grafted conditions, with distinct responses observed between grafting regimes.

To enable direct comparisons, networks were constructed using the top 100 edges with the highest correlation coefficients (|*r*| > 0.8, *p* < 0.05) for each treatment. This standardization ensures that observed differences in network metrics reflect genuine changes in interaction patterns rather than network size variations.

Under grafted conditions, the companion cropping treatments exhibited higher network densities than CK. The average clustering coefficient was significantly higher in T1, T2, and T4 relative to CK, whereas no significant difference was observed for T3. With the number of edges standardized to 100 across all treatments, the total number of nodes decreased consistently under companion cropping. Notably, the proportions of fungal nodes increased in companion treatments relative to CK, accompanied by corresponding decreases in bacterial nodes. Average path length was significantly shorter in T1 and T2 than in CK, whereas significantly longer in T3 and T4. Interaction sign analysis revealed that T2 and T3 harbored higher proportions of positive correlations (and lower negative correlations) than CK, whereas T1 and T4 showed the inverse pattern, with reduced positive and increased negative correlations (Figure 5).

Under non-grafted conditions, companion cropping uniformly increased network density but significantly decreased clustering coefficients relative to CK. Node numbers declined in companion cropping treatments under standardized edge constraints. In contrast to grafted systems, companion cropping reduced the proportion of fungal nodes while increasing the proportion of bacterial nodes. All treatments exhibited significantly longer average path lengths than CK, and all treatments (except T3) had a higher proportion of positive correlations (Figure 5).

### 3.4 Effects of grafting and companion cropping on watermelon yield and fruit quality

**Figure 6:**
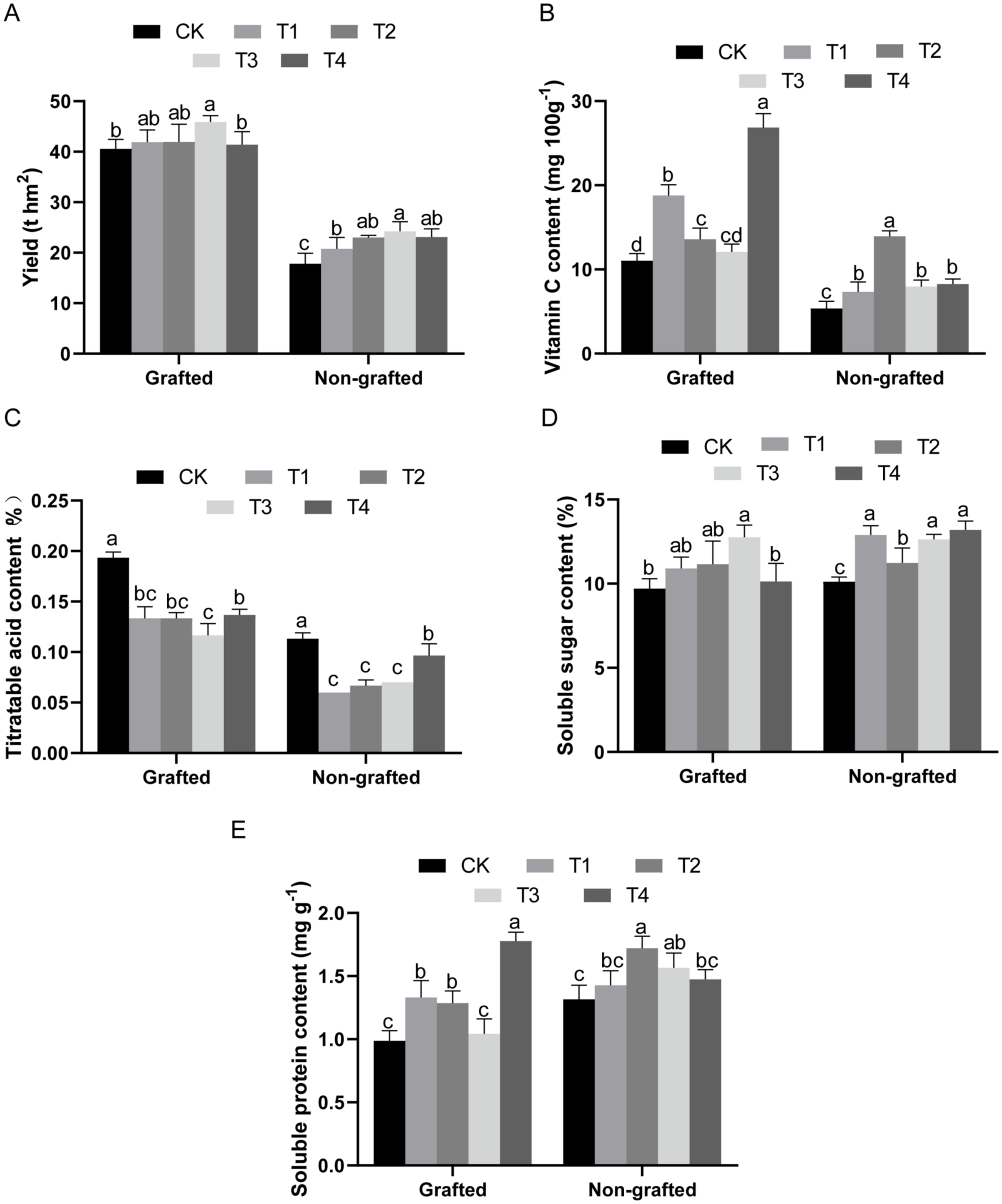
Effects of different treatments on yield and quality of watermelon. **(A)** Yield. **(B)** Vitamin C content. **(C)** Titratable acid content. **(D)** Soluble sugar content. **(E)** Soluble protein content. Data are expressed as mean ± standard deviation. Values with different lowercase letters indicate significant differences among the five companion cropping treatments within the same grafting level (*p* < 0.05).

Grafting significantly affected yield and fruit quality traits (Figure 6). Compared with non-grafted plants, grafting increased yield (Figure 6A), vitamin C content (Figure 6B) and titratable acidity (Figure 6C) by 94.25, 77.40, and 92.64%, respectively (*p* < 0.05). Conversely, grafting significantly reduced soluble sugar (Figure 6D) and soluble protein contents (Figure 6E; *p* < 0.05) by 10.66 and 14.31%, respectively (*p* < 0.05).

The effects of companion cropping were strongly dependent on grafting status. Under grafted conditions, none of the treatments significantly affected yield, except T3 (Figure 6A); however, soluble protein and vitamin C contents were significantly elevated across the companion treatments (Figures 6B,E; *p* < 0.05). Soluble sugar content increased under companion cropping, although statistical significance was attained only in T3 (Figure 6D; *p* < 0.05). Under non-grafted conditions, the companion cropping treatments significantly improved yield, vitamin C, soluble sugar, and soluble protein contents (*p* < 0.05; Figures 6A,B,D,E). Notably, regardless of grafting status, titratable acidity was consistently and significantly lower across the companion cropping treatments relative to CK (Figure 6C; *p* < 0.05).

### 3.5 Relationships between root Endophytic microbial communities and watermelon yield and fruit quality

#### 3.5.1 Redundancy analysis (RDA)

**Figure 7:**
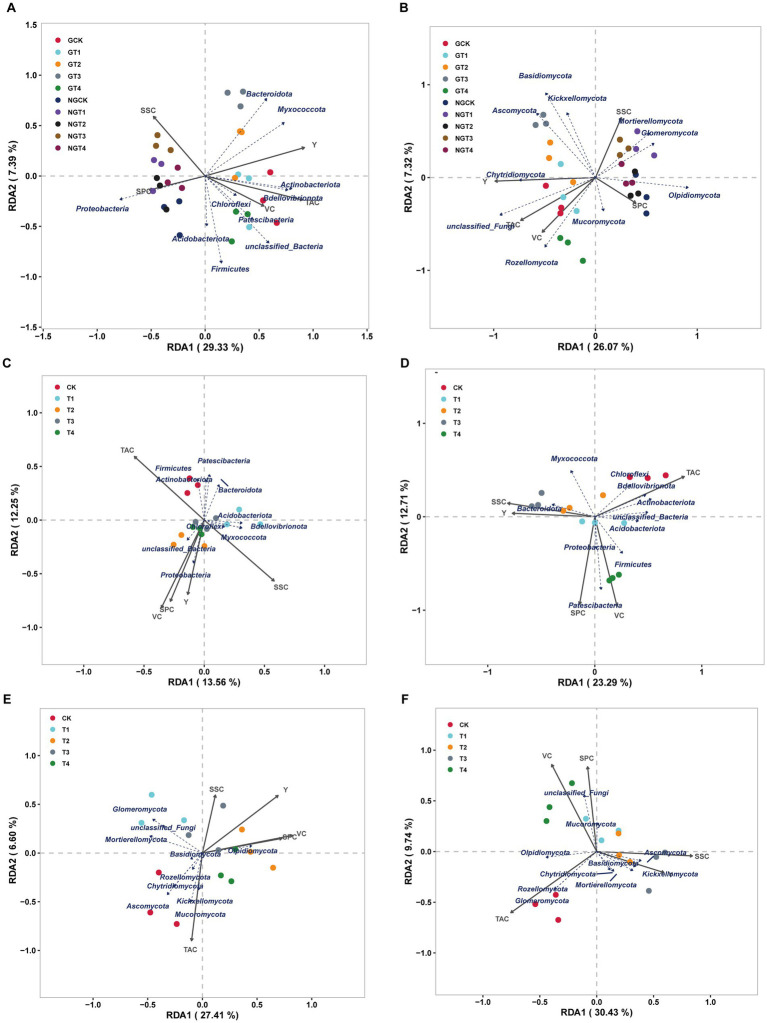
Redundancy analysis (RDA) of root endophytic microbial communities and watermelon yield and fruit quality traits. **(A)** Bacteria across all treatments. **(B)** Fungi across all treatments. **(C)** Bacteria under grafted conditions. **(D)** Fungi under grafted conditions. **(E)** Bacteria under non-grafted conditions. **(F)** Fungi under non-grafted conditions.

Redundancy analysis (RDA) was conducted on bacterial and fungal community datasets (top 10 phyla by relative abundance) alongside fruit yield and quality traits, to reveal the relationships between watermelon root endophytic microbial communities and yield and fruit quality. The first two RDA axes explained 36.72% of the total variance for bacterial communities and 33.39% of the total variance for fungal communities across all treatments, respectively. Grafted and non-grafted samples clearly separated along the RDA1 axis (Figures 7A,B).

Under grafted conditions, for bacteria, the first two axes explained 36.00% of total variance. Actinobacteriota, Bdellovibrionota and Chloroflexi dominated in CK and showed positive correlations with titratable acidity. In contrast, T2 and T3 were enriched in Bacteroidota and Myxococcota, both positively correlated with yield and soluble sugar content. Meanwhile, T4 harbored abundant Proteobacteria, Firmicutes and Patescibacteria; these phyla were positively associated with yield, vitamin C and soluble protein content, but negatively correlated with soluble sugar content and titratable acidity (Figure 7C). For fungi, the first two axes explained 40.17% of the total variance. Glomeromycota, Rozellomycota and Chytridiomycota predominated in CK, exhibiting positive correlations with titratable acidity and negative correlations with soluble sugar and soluble protein contents. By contrast, T2 and T3 were dominated by Ascomycota, Basidiomycota and Mortierellomycota, which positively correlated with yield and soluble sugar content. Notably, T1 and T4 accumulated Mucoromycota and unclassified_fungi, which were positively linked to vitamin C and soluble protein content, while Olpidiomycota, also enriched in T4, showed a positive correlation with vitamin C (Figure 7D).

Under non-grafted conditions, for bacteria, the first two axes explained 25.81% of the total variance. Firmicutes, Actinobacteriota and Patescibacteria prevailed in CK; these phyla were positively correlated with titratable acidity but negatively correlated with yield, vitamin C, soluble protein and soluble sugar contents. In contrast, T1 was dominated by Bacteroidota, Bdellovibrionota and Myxococcota: Bacteroidota correlated positively with titratable acidity, whereas Bdellovibrionota and Myxococcota were positively associated with soluble sugar content and negatively associated with titratable acidity. Notably, T2–T4 were enriched in Proteobacteria, unclassified_bacteria and Chloroflexi, which displayed positive correlations with yield, vitamin C and soluble protein contents and a negative correlation with titratable acidity (Figure 7E). For fungi, the first two axes explained 34.01% of the total variance. Mucoromycota, Rozellomycota, Chytridiomycota, Ascomycota and Basidiomycota were dominant in CK, positively correlated with titratable acidity but negatively correlated with yield, vitamin C, soluble protein and soluble sugar contents. In contrast, T1 was enriched in Glomeromycota and unclassified_fungi, both positively associated with soluble sugar content. Notably, Olpidiomycota dominated T2 and correlated positively with yield, soluble sugar, vitamin C and soluble protein content. Similarly, in T3, Glomeromycota and unclassified_fungi showed positive associations with soluble sugar content, while Olpidiomycota correlated positively with yield, soluble sugar, vitamin C and soluble protein contents. In T4, Olpidiomycota maintained positive correlations with yield, soluble sugar, vitamin C and soluble protein contents, whereas Kickxellomycota and Mucoromycota were positively linked to titratable acidity (Figure 7F).

Collectively, these RDA results preliminarily revealed directional associations between dominant microbial phyla and yield and fruit quality traits under different cultivation regimes. Subsequent Pearson correlation analysis was conducted to further quantify the magnitude and statistical significance of pairwise phylum–trait correlations for validation.

#### 3.5.2 Phylum-level correlations

Pearson’s correlation analysis identified statistically robust associations between dominant endophytic bacterial and fungal phyla and key yield and quality traits, regardless of grafting treatment.

**Figure 8:**
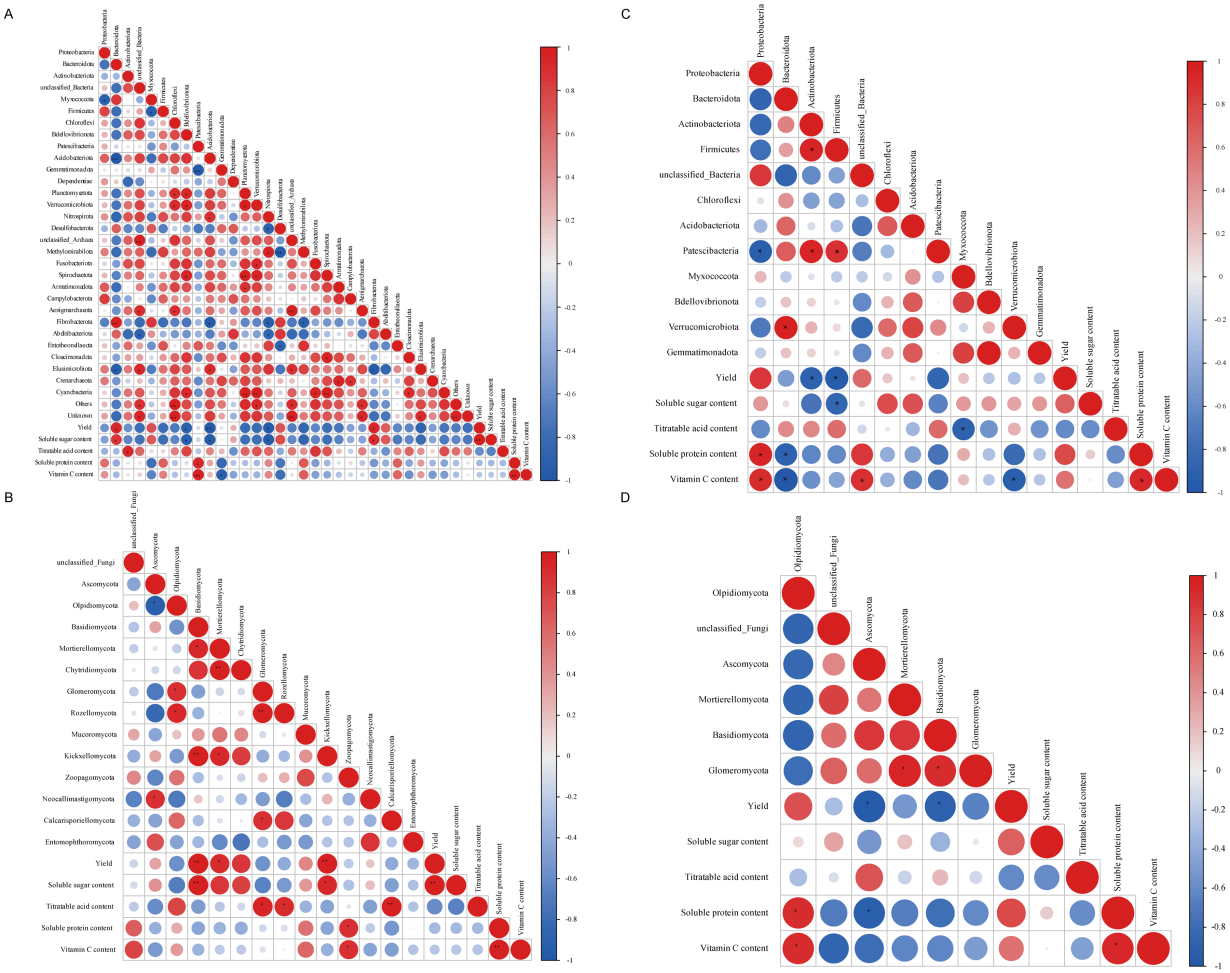
Correlation between root endophytes and yield and fruit quality of watermelon at the phylum level. **(A)** Bacteria in grafted watermelon. **(B)** Fungi in grafted watermelon. **(C)** Bacteria in non-grafted watermelon. **(D)** Fungi in non-grafted watermelon. The yield and fruit quality were consistent for grafted watermelon **(A,B)** and non-grafted watermelon **(C,D)**. *, **, and *** denote significant differences at the *p* < 0.05, *p* < 0.01, and *p* < 0.001 levels, respectively.

Under grafted conditions, fruit yield was significantly positively correlated with soluble sugar content(*p* < 0.01), as were soluble protein and vitamin C contents (Figure 8A; *p* < 0.01).

At the bacterial phylum level, Bacteroidota and Fibrobacterota showed significant positive correlations with yield and soluble sugar content (Figure 8A; *p* < 0.05). In contrast, Acidobacteriota, Nitrospirota and Bdellovibrionota were significantly negatively correlated with both traits (*p* < 0.05). Actinobacteriota was significantly positively correlated with titratable acidity (*p* < 0.05). Patescibacteria showed strong positive correlations with soluble protein and vitamin C contents (*p* < 0.05), whereas Gemmatimonadota was significantly negatively correlated with vitamin C content (*p* < 0.05).

Among fungal phyla, Basidiomycota and Kickxellomycota were significantly positively correlated with yield and soluble sugar content (Figure 8B; *p* < 0.05). Mortierellomycota was significantly positively correlated with soluble protein content (*p* < 0.05). Zoopagomycota showed significant positive correlations with both soluble protein and vitamin C contents (*p* < 0.05).

Under non-grafted conditions, soluble protein content was significantly positively correlated with vitamin C content (Figures 8C,D; *p* < 0.05). At the bacterial phylum level, Proteobacteria was significantly positively correlated with soluble protein content (*p* < 0.05), and unclassified_Bacteria was significantly positively correlated with vitamin C content (Figure 8C; *p* < 0.05). Conversely, Bacteroidota was significantly negatively correlated with soluble protein content (*p* < 0.05), and Verrucomicrobiota was significantly negatively correlated with vitamin C content (*p* < 0.05). Actinobacteriota and Firmicutes were significantly negatively correlated with yield (*p* < 0.05), while Firmicutes was significantly positively correlated with soluble sugar content (*p* < 0.05). Myxococcota was significantly negatively correlated with titratable acidity (*p* < 0.05).

At the fungal phylum level, Olpidiomycota showed significant positive correlations with soluble protein and vitamin C contents (Figure 8D; *p* < 0.05). Ascomycota and Basidiomycota were significantly negatively correlated with yield (*p* < 0.05), while Ascomycota also had a significant negative correlation with soluble protein content (*p* < 0.05).

#### 3.5.3 Genus-level correlations

Pearson’s correlation analysis combined with hierarchical clustering revealed robust, condition-dependent associations between dominant endophytic bacterial and fungal genera in watermelon roots and key yield and fruit quality, irrespective of grafting treatment. Clustering results further highlighted functional coherence among co-clustering taxa, underscoring the divergent regulatory roles of root endophytic microbial communities in watermelon yield and quality formation under different grafting conditions. Under grafted conditions, fruit yield and soluble sugar content, as well as soluble protein and vitamin C contents, exhibited significant positive correlations (Supplementary Figures S1A,B; *p* < 0.05).

At the bacterial genus level, *Flavobacterium*, *Pseudoflavitalea* and *Dyadobacter* were significantly positively correlated with fruit yield (Supplementary Figure S1A; *p* < 0.05). *Dyadobacter* also showed a significant positive correlation with soluble sugar content (*p* < 0.05), whereas *Methylobacillus* and *Mesorhizobium* were significantly negatively correlated with this trait (*p* < 0.05). *Bacillus* and *Pseudoxanthomonas* displayed significant positive correlations with soluble protein content (*p* < 0.05), and *Pseudoxanthomonas* was further significantly positively correlated with vitamin C content (*p* < 0.05). In contrast, the “Others” taxonomic group exhibited a significant negative correlation with vitamin C content (*p* < 0.05).

At the fungal genus level under grafted conditions, *Plectosphaerella*, *Penicillium*, unclassified_*Basidiomycota*, *Podospora*, *Candida*, unidentified and *Triangularia* were significantly positively correlated with both fruit yield and soluble sugar content (Supplementary Figure S1B; *p* < 0.05). *Mortierella* was significantly positively associated with fruit yield (*p* < 0.05). *Fusicolla*, *Tuber*, *Aspergillus*, and *Millerozyma* showed significant positive correlations with titratable acidity (*p* < 0.05). *Dactylonectria* was significantly positively correlated with soluble protein and vitamin C contents (*p* < 0.05), whereas *Saitozyma* exhibited significant negative correlations with both traits (*p* < 0.05). Hierarchical clustering further revealed functional consistency among co-clustering taxa. *Pseudoflavitalea* was consistently associated with fruit yield, soluble protein, and vitamin C contents, while *Dyadobacter* was strongly correlated with both fruit yield and soluble sugar content. Notably, *Plectosphaerella*, *Penicillium*, unclassified *Basidiomycota*, *Podospora*, *Candida*, and unclassified_Fungi formed a cohesive cluster linked to fruit yield and soluble sugar content, and *Dactylonectria* and *Saitozyma* occupied opposing positions within a subcluster enriched for associations with soluble protein and vitamin C contents.

Under non-grafted conditions, soluble protein content was significantly positively correlated with vitamin C content (Supplementary Figures S2A,B; *p* < 0.05).

At the bacterial genus level, the [*Rhizobium*]*_sphaerophysae* group was significantly positively correlated with fruit yield (Supplementary Figure S2A; *p* < 0.05), whereas *Bacillus*, *Pseudoxanthomonas*, *Streptomyces*, *Cellvibrio*, *Lysobacter*, *Pseudomonas*, *Massilia*, and *Paenibacillus* were significantly negatively correlated with fruit yield (*p* < 0.05). *Ensifer* showed a significant negative correlation with soluble sugar content (*p* < 0.05). *Paucibacter* exhibited significant positive correlations with both soluble protein and vitamin C contents (*p* < 0.05), while *Flavobacterium*, the *Allorhizobium–Neorhizobium–Pararhizobium–Rhizobium* complex, and *Sphingomonas* were significantly negatively correlated with these two traits (*p* < 0.05). The “Others” group and *Hydrogenophaga* were significantly negatively correlated with soluble protein content (*p* < 0.05).

At the fungal genus level under non-grafted conditions, *Olpidium* (belonging to Olpidiomycota) showed significant positive correlations with soluble protein and vitamin C contents (Supplementary Figure S2B; *p* < 0.05), whereas *Thermomyces* exhibited significant negative correlations with both traits (*p* < 0.05). *Monosporascus*, *Cladosporium*, *Aspergillus*, *Plectosphaerella*, *Toxicocladosporium*, unclassified *Dothideomycetes*, and the “Others” group were significantly negatively correlated with fruit yield (*p* < 0.05). *Aureobasidium* was significantly negatively correlated with soluble sugar content (*p* < 0.05). The “Others” group also displayed a strong negative correlation with soluble protein content (*p* < 0.05). Cluster analysis indicated that *Paucibacter*, *Olpidium*, and *Thermomyces* formed a cluster associated with soluble protein and vitamin C contents, with opposite correlation directions.

## 4 Discussion

### 4.1 Effects of grafting and companion cropping on Endophytic microbial community diversity, composition and co-occurrence networks in watermelon roots

Endophytic microbial communities are more abundant and functionally diverse in roots than in aerial tissues, where they contribute to nutrient acquisition, stress tolerance, and pathogen suppression (Chen X. F. et al., 2024; Andrade et al., 2023; Jorge et al., 2025). Grafting and companion cropping are widely used agroecological strategies that alleviate continuous monocropping constraints, including nutrient depletion and microbial homogenization, while enhancing microbial diversification and interspecific interactions (Shi et al., 2026; Li et al., 2024).

This study demonstrates that grafting exerts a pronounced and taxon-specific regulatory influence on the endophytic microbiota of watermelon roots. Specifically, grafting significantly increased OTU richness and *α*-diversity indices for both bacterial and fungal endophytes; however, it enhanced the Shannon diversity in bacterial communities while reducing it in fungal communities. This divergence between richness and diversity in fungal communities warrants particular attention. The observed increase in fungal richness suggests that grafting facilitates the colonization of a broader array of fungal taxa. This is likely achieved through rootstock-mediated modifications of root exudation profiles and enhanced carbon allocation to the rhizosphere (Qiao et al., 2024). The concomitant reduction in Shannon and Simpson indices, however, indicates that this expanded colonization is accompanied by a decline in community evenness, with certain fungal taxa achieving dominance at the expense of others.

This pattern is consistent with a “selection effect” imposed by the grafted rootstock. The physiological integration of rootstock and scion may create selective pressures that favor specific fungal lineages, particularly those with beneficial functions, such as nutrient mobilization, stress mitigation, and pathogen antagonism, while excluding or inhibiting others, including opportunistic or pathogenic saprotrophs (Qiao et al., 2024). The observed reduction in fungal diversity reflects directional filtering of root fungi under grafting conditions, which is supported by our LEfSe, RDA and correlation analyses. Previous studies suggested that grafted crops exhibit enhanced disease suppression through competitive exclusion of phytopathogens such as *Fusarium* spp. (Keinath and Hassell, 2014; Ge et al., 2022). However, our genus-level data reveal inconsistent responses of *Fusarium*. This genus dominates both grafted and non-grafted root endophytes. Its abundance drops only under T1–T3 in non-grafted roots and rises under T4, and no consistent suppression is observed across grafted treatments. Grafting continuously suppresses the pathogenic genus *Olpidium* under T1–T3, while the root rot pathogen *Monosporascus* accumulates under T1 and T2. These findings corroborate those of Qiao et al. (2024), who reported the grafting-induced restructuring of the watermelon rhizosphere microbiome toward a more disease-suppressive configuration, reinforcing the interpretation that grafting exerts targeted, functionally consequential modulation of root endophytic microbial communities, suppressing specific pathogens such as *Olpidium* while tolerating or even enriching others like *Monosporascus* under certain treatments, rather than broad inhibition of all pathogenic fungal taxa.

Companion cropping significantly reduced OTU richness and Chao1 diversity in both bacteria and fungi under grafted and non-grafted conditions, with the strongest suppression in T2. These reductions likely result from competition for nutrients and carbon, as well as allelopathic interference limiting niche colonization by rare or specialist taxa (Su et al., 2025; Kong et al., 2025). The strong treatment dependence indicates that companion cropping effects are highly species-specific. Relevant trials of Allium companion cropping confirmed that co-cultivation with Chinese chives significantly lowered the Chao1, Shannon and Simpson indices of both rhizosphere and endophytic bacteria in pepper. Allelochemicals secreted by Allium roots exert directional filtering pressure on the microbial communities of co-cultivated crops (Sun L. et al., 2025; Sun N. et al., 2025). Research on root endophytes within peanut–maize intercropping systems further demonstrates that interspecific interactions modulate microbiota in a microhabitat- and host-dependent manner. Rhizosphere soil and root endophytic communities exhibit entirely divergent responses to intercropping, as intercropping reduces the endophytic OTU richness and diversity of peanut roots while elevating those of maize roots (Chen et al., 2022). Moreover, grafting intensified the suppressive effects of companion cropping, suggesting synergistic regulation of microbial assembly (Ezazi et al., 2021). This indicates that grafting and companion cropping jointly shape microbial communities through direct root physiological effects and indirect soil-mediated interactions, with microbial interspecies relationships acting as key mediators (Vink et al., 2021; Yang et al., 2024).

LEfSe analysis revealed significantly different indicator taxa between grafted and non-grafted systems. For bacteria, under grafted conditions, the lowest discriminative taxonomic levels were primarily resolved at the phylum level, encompassing Bacteroidetes (CK), Firmicutes (T1), Chloroflexi (T2), Proteobacteria (T3), and Actinobacteria (T4). These phyla are known to play important roles in nutrient cycling and plant-microbe interactions (Wang et al., 2021; Huang et al., 2023). In non-grafted systems, however, these phylum-level associations shifted markedly: no phylum-level indicators were detected for T1 and T3, whereas numerous differentially abundant clades were identified at the genus and species levels, suggesting that the non-grafted condition primarily modulates bacterial community structure via low-abundance taxa. For fungi, signature taxa under grafting conditions were exclusively confined to Ascomycota. CK, T1, T2, T3, and T4 were characterized by Cladosporium, Penicillium, Plectosphaerella, Hypomyces, and Monosporascus, respectively; these genera encompass functions ranging from biological control (Cladosporium, Penicillium) to nutrient cycling (Plectosphaerella) and soil-borne pathogenesis (Monosporascus cannonballus) (Taheri et al., 2022; Sales et al., 2010). This phenomenon originates from the directional screening and enrichment of specific microbial taxa under Allium companion cropping. Nishioka et al. (2019) proposed that the disease-suppressive effects of Allium crops are closely associated with the selective accumulation of antagonistic Flavobacterium, rather than broad-spectrum inhibition of microorganisms. Such directional microbial selection means the regulatory effects within companion cropping systems are jointly constrained by crop combinations and cultivation environments. The Chinese chive–pepper companion cropping trial conducted by Sun L. et al. (2025) and Sun N. et al. (2025) also supports the context-dependent responses of microbial communities. The enrichment of pathogenic Monosporascus under T4 treatment in this study further demonstrates that companion cropping is not a universal approach to optimize beneficial microbiota, and its microbial regulatory effects display prominent treatment specificity. By contrast, the non-grafted system was typified by Olpidiomycota and Mortierellomycota, taxa frequently associated with saprotrophic and stress-tolerance functions (Van der Heijden et al., 2008). Notably, within the non-grafted system, T3 lacked phylum-level fungal indicators, with significant differentiation emerging only at the genus and species levels, suggesting that this treatment primarily restructures the fungal community through rare taxa modulation. Collectively, these LEfSe results complement the community-level compositional analyses, pinpoint specific taxa driving treatment divergence, and provide a taxonomic foundation for interpreting the co-occurrence network dynamics discussed below.

PCoA based on Bray–Curtis dissimilarity confirmed that companion cropping significantly altered microbial community composition. To statistically validate the patterns observed in the PCoA ordination, PERMANOVA confirmed that grafting, companion cropping, and their interaction each significantly influenced root endophytic microbial community structure (*p* < 0.001). These compositional shifts were weaker under grafted conditions, suggesting that rootstocks buffer microbial responses by stabilizing root exudation profiles and strengthening host-imposed filtering (Liu D. Y. et al., 2025). In contrast, non-grafted plants showed stronger compositional shifts, indicating higher microbiome plasticity. These results extend previous observations from rhizosphere studies to the endophytic compartment (Yang, et al., 2024; Chen, et al., 2022).

Both grafting and companion cropping significantly altered microbial composition at phylum and genus levels. At the bacterial level, grafted plants harbored more bacterial phyla than non-grafted plants, indicating enhanced microbial recruitment capacity. Dominant bacterial phyla included Proteobacteria, Actinobacteriota, Bacteroidota, and Firmicutes. Although these taxa were consistently present, their relative abundances varied significantly across treatments, reflecting ecological plasticity (Xu et al., 2020). Proteobacteria, a dominant plant-associated phylum involved in carbon cycling and nutrient mobilization (Bian et al., 2021; Wu et al., 2021), increased under companion cropping, consistent with enhanced nutrient turnover and microbial activity (PérezIzquierdo et al., 2019; Jiang et al., 2019). By contrast, Actinobacteriota, another prevalent bacterial phylum in soil and root niches, contributes to plant health through biocontrol activity and nutrient transformation, ultimately enhancing stress tolerance, yield, and fruit quality (Kinsey et al., 2026; Wang et al., 2021). Similarly, Bacteroidota, commonly enriched in rhizosphere environments, participates in the degradation of complex organic substrates and mediates carbon and nitrogen cycling, thereby influencing microbial community stability and plant nutrient acquisition (Huang et al., 2023; Kruczyńska et al., 2023). However, contrary to previous companion cropping studies (Liu et al., 2024), companion cropping in the present study significantly reduced the relative abundances of both Bacteroidota and Actinobacteriota under both grafted and non-grafted conditions. This discrepancy may be attributed to differences in companion crop species or cropping system configurations, potentially involving the allelopathic effects of chives and onions that selectively inhibit these phyla. This hypothesis warrants further investigation using metabolomic or metatranscriptomic approaches.

At the fungal level, dominant phyla included Ascomycota, Olpidiomycota, and unclassified fungi. Ascomycota is the most phylogenetically diverse and widely distributed fungal phylum, with members engaging in multifaceted interactions with crop plants, ranging from pathogenesis to mutualism and ecological regulation, that exert dual effects on plant performance and agroecosystem stability (Van der Heijden et al., 2008). Notably, grafting status modulated the response of Ascomycota to companion cropping. Under grafted conditions, the companion treatments, except T4, significantly increased its abundance, whereas under non-grafted conditions, companion cropping consistently reduced its abundance. This contrasting pattern suggests that grafting can fundamentally alter the direction of companion cropping-induced shifts in fungal community composition. This finding diverges from previous studies that examined grafting or companion cropping independently (Zhou et al., 2024; Liu et al., 2020), and further underscores the interactive nature of these two agricultural interventions in shaping root endophytic microbial communities.

Beyond compositional shifts, microbial co-occurrence networks provide insight into interaction dynamics among root endophytes. In such networks, positive correlations are generally interpreted as indicative of cooperative or synergistic interactions among taxa, whereas negative correlations typically reflect competitive or antagonistic relationships (Zheng et al., 2018; Durga et al., 2026). This study revealed that grafting exerts a decisive modulatory effect on how companion cropping reshapes the topological architecture and interaction dynamics of root endophytic microbial community networks in watermelon roots. Under grafted conditions, companion cropping consistently enhanced network density, the average clustering coefficient, and the proportion of fungal nodes. These changes indicate a shift toward more interconnected and fungus-enriched networks, suggesting enhanced structural complexity and tighter microbial associations.

The directions and magnitudes of these regulatory effects varied markedly across companion cropping treatments. Specifically, T2 and T3 exhibited significantly higher proportions of positive correlations, and correspondingly lower proportions of negative correlations, than CK, implying a net attenuation of interspecific competition and a functional shift toward cooperative microbial associations (Hernandez et al., 2021; Romdhane et al., 2022). In contrast, T1 and T4 exhibited the inverse trend, with lower positive and higher negative correlations, which can be potentially attributed to species-specific incompatibilities, such as allelopathic interference, root exudate mismatch, or niche overlap, between the companion crops and watermelon under grafted conditions. This observation aligns with reports of crop-dependent competitive outcomes in apple-based agroforestry systems (Zhao et al., 2024).

Under non-grafted conditions, companion cropping increased network density but reduced clustering coefficient and bacterial network dominance. Although bacterial node numbers increased the decline in clustering and the increase in average path length suggest that these additional taxa were weakly integrated into the network structure. This indicates that microbial recruitment occurred without corresponding increases in interaction stability or complexity. In a long-term monocropping context, and in the absence of rootstock-mediated regulation, these recruited bacteria are likely opportunistic or transient taxa that do not establish stable cooperative relationships (Cao et al., 2024). As a result, the network becomes more dispersed and less cohesive despite higher taxonomic richness.

Notably, most companion treatments under non-grafted conditions still showed higher proportions of positive correlations than CK (except T3), indicating that companion cropping generally promotes a baseline level of microbial cooperation even without grafting support. Among all treatments, T2 and T3 consistently exhibited the strongest cooperative network features, characterized by higher positive correlation ratios and greater modular organization. This suggests that these treatments provide more favorable ecological conditions for stable and synergistic microbial interactions in grafted watermelon systems.

Overall, these findings demonstrate that appropriately designed companion cropping regimes, particularly when integrated with grafting, can systematically reconfigure the interaction landscape of watermelon root endophytes, shifting the balance from competition toward cooperation. The pronounced divergence in network topology between grafted and non-grafted treatments further implies fundamentally distinct community assembly mechanisms under these management regimes (Yang et al., 2020). Critically, such microbiome-level restructuring represents a plausible mechanistic pathway through which companion cropping, especially in combination with grafting, modulates watermelon growth, physiological performance, and yield formation (Chen M. F. et al., 2024).

### 4.2 Grafting and companion cropping effects on watermelon yield and fruit quality

A growing body of evidence indicates that both grafting and companion cropping profoundly reshape the composition and functional potential of root endophytic microbial communities (Yang et al., 2024; Wang et al., 2022). This microbiome-mediated pathway represents a key mechanism through which these agronomic interventions indirectly regulate crop yield and fruit quality. Consistent with this framework, the present study demonstrated that grafting and companion cropping exert distinct yet complementary effects on watermelon yield and key fruit quality parameters. Grafting significantly enhanced yield, titratable acidity, and vitamin C content, while concurrently reducing soluble sugar and soluble protein contents. These coordinated shifts suggest that rootstock selection influences carbon and nitrogen partitioning in the scion, preferentially allocating photosynthetic assimilates toward organic acid and ascorbic acid biosynthesis at the expense of soluble carbohydrate and protein accumulation (Takgoa et al., 2025; Liu X. N. et al., 2025). Notably, this pattern contrasts with findings reported by Shrestha et al. (2022), who observed improved muskmelon yield without adverse effects on fruit quality following grafting. Such divergence likely arises from interspecific differences in sink strength and metabolic regulation, as well as variations in rootstock–scion compatibility, environmental conditions, or cultivation duration. These factors merit rigorous comparative analyses in future works.

Importantly, companion cropping consistently improved watermelon yield and elevated soluble sugars, soluble proteins, and vitamin C contents across both grafted and non-grafted systems, while significantly lowering titratable acidity relative to CK. This consistent enhancement of yield and nutritional quality, coupled with a reduction in acidity, suggests that companion cropping promotes a more balanced fruit metabolite profile, potentially through improved nutrient acquisition, rhizosphere microbiome modulation, or stress mitigation. This finding is congruent with observations in tomato companion cropping systems (He et al., 2025) and reinforces the hypothesis that companion cropping confers broadly conserved benefits for horticultural crop performance and fruit nutritional value.

Overall, these findings suggest that grafting primarily modulates internal physiological allocation patterns, whereas companion cropping enhances external resource acquisition and microbe-mediated nutrient regulation, together shaping yield and fruit quality outcomes in distinct but complementary ways.

### 4.3 Correlations between root endophytic microbial communities and watermelon yield and fruit quality

Redundancy analysis (RDA) and Pearson correlation analysis were integrated to investigate the associations between root endophytic microbial community composition and watermelon yield and fruit quality. RDA results revealed that the first two canonical axes accounted for 36.72 and 33.39% of the total variation in bacterial and fungal community structures, respectively. Although the cumulative explained variance was moderate, this magnitude is consistent with the typical ranges reported for root endophyte studies (Brigham et al., 2023). The substantial unexplained variation observed in the present study could be attributed to latent biotic and abiotic drivers, including root exudate profiles, phytohormonal dynamics, dispersal limitation, and stochastic microbial assembly processes (Lailheugue et al., 2024; Li et al., 2023). Clear spatial separation between grafted and non-grafted samples along the RDA1 axis identified grafting as a primary deterministic driver governing the assembly and structural divergence of root endophytic communities, which is consistent with previous findings (Ling et al., 2015; Wang et al., 2022). Furthermore, distinct clustering of samples from different cultivation treatments in the ordination space indicated that cultivation management exerted stable and non-random modulation on the structure of root endophytic microbial communities. Pearson correlation analysis further quantified the linear relationships between the relative abundances of dominant microbial phyla and watermelon yield and fruit quality traits under different cultivation regimes, which helps screen key microbial taxa responsible for regulating watermelon yield formation and quality improvement.

At the phylum level, under grafted conditions, the abundances of Bacteroidota and Fibrobacterota were significantly positively correlated with marketable yield and soluble sugar content. This identified them as core beneficial phyla for high yield and superior quality in grafted watermelon. These taxa may promote yield formation and sugar accumulation through multiple ecological functions, including the mineralization of rhizosphere organic matter, activation of soil nutrients (e.g., phosphorus and micronutrients), and regulation of host carbon allocation to sink tissues (Lv et al., 2025).

In contrast, Acidobacteriota, Nitrospirota, and Bdellovibrionota showed significant negative correlations with yield and soluble sugar content, suggesting inhibitory roles, potentially through competition with beneficial microbes, disruption of carbon–nitrogen balance, or induction of stress responses that divert resources from fruit development (Komal et al., 2025).

Specialized associations were also observed. Actinobacteriota abundance was significantly positively correlated with titratable acidity, indicating a role in maintaining fruit flavor balance (Wang et al., 2020). Patescibacteria showed significantly positive correlations with soluble protein and vitamin C contents, supporting its contribution to grafting-mediated improvement in fruit nutritional quality (Tian et al., 2024).

Among fungi, the abundances of Basidiomycota and Kickxellomycota were significantly positively correlated with yield and soluble sugar content, suggesting growth promoting functions in grafted systems (Liu et al., 2026). Glomeromycota (arbuscular mycorrhizal fungi) showed positive correlations with titratable acidity, while Rozellomycota and Zoopagomycota correlated with vitamin C synthesis, and soluble protein accumulation, respectively. These patterns indicate functional specialization among fungal phyla in organic acid regulation and nutrient biosynthesis, consistent with Tan et al. (2026).

Under non-grafted conditions, the functional characteristics of the root endophytic microbiome exhibited marked differentiation. Proteobacteria abundance was significantly positively correlated with soluble protein and vitamin C contents, indicating a key role for this phylum in maintaining fruit nutritional quality in the absence of grafting. Conversely, Bacteroidota and Verrucomicrobiota showed significant negative correlations with these nutritional indicators, suggesting context-dependent antagonism. This pattern contrasts with the beneficial associations observed for these phyla under grafted conditions, highlighting the strong influence of rootstock–scion interactions on microbial functional expression. These findings differ from those of Chen et al. (2026), who observed that both Proteobacteria and Bacteroidota are negatively correlated with grain protein content in wheat. This discrepancy may be attributed to factors such as crop species and cultivation conditions. The underlying mechanisms warrant further investigation.

Actinobacteriota showed a negative correlation with yield under non-grafted conditions, contrasting with its positive association with titratable acidity under grafting, further confirming grafting-induced functional reprogramming of this phylum (Zhang et al., 2025; Liu M. et al., 2025). Firmicutes exhibited a dual role, being positively correlated with soluble sugar but negatively correlated with yield. This suggests that while members of this phylum may enhance sugar metabolism and transport (Hemkemeyer et al., 2024; Pang et al., 2020; Ji et al., 2025), they may also impair overall plant productivity under continuous monocropping stress by affecting root function and nutrient uptake (Li et al., 2025).

Among fungi, Olpidiomycota was positively correlated with soluble protein and vitamin C, likely due to cellulolytic activity enhancing nutrient availability (Boer et al., 2005). In contrast, Ascomycota and Basidiomycota were negatively correlated with yield, while Ascomycota also negatively correlated with soluble protein. These patterns suggest that under non-grafted monocropping stress, these phyla may shift toward saprophytic or pathogenic roles, contributing to reduced plant performance (Sun L. et al., 2025; Sun N. et al., 2025; Wang et al., 2025; Wang et al., 2024).

## 5 Conclusion

The present study demonstrates that grafting and companion cropping act synergistically to reshape the structures of root endophytic microbial communities in watermelon under long-term continuous cropping, thereby regulating fruit yield and quality. Grafting significantly increased the richness of root endophytic bacteria and fungi, shifted community composition and co-occurrence networks, and improved marketable yield, titratable acidity, and vitamin C content, while decreasing soluble sugar and soluble protein concentrations. Although companion cropping decreased overall microbial richness, it effectively restructured community composition and interspecific interaction patterns, and its effects on yield and quality were grafting-dependent. Importantly, irrespective of grafting status, companion cropping significantly reduced fruit titratable acidity, a response closely associated with microbial community restructuring. Among the treatments, T2 and T3 exhibited the strongest effects, driving microbial interactions from competition toward cooperation. These findings confirm that the root endophytic microbiome acts as a crucial biological pathway mediating the impacts of agronomic practices on crop performance under continuous monocropping systems. However, given the limited sample size, the network analysis results should be interpreted as preliminary, and further validation through larger-scale studies is warranted.

## Data Availability

The raw sequencing data have been deposited in the NCBI SRA under BioProject accession number PRJNA1462526.

## Footnotes

1
https://international.biocloud.net/zh/user/login

2
https://cloud.metware.cn/#/home

## Supplementary material

SUPPLEMENTARY FIGURE S1 Correlation between root endophytes and yield and quality of watermelon at the genus level in grafted watermelon. **(A)** Bacterial community and **(B)** fungal community. *, **, and *** denote significant differences at the *p* < 0.05, *p* < 0.01, and *p* < 0.001 levels, respectively.

SUPPLEMENTARY FIGURE S2 Correlation between root endophytes and yield and quality of watermelon at the genus level in non-grafted watermelon. **(A)** Bacterial community and **(B)** fungal community. *, **, and *** denote significant differences at the *p* < 0.05, *p* < 0.01, and *p* < 0.001 levels, respectively.
